# Evaluation of Toxicant-Associated Fatty Liver Disease and Liver Neoplastic Progress in Sprague-Dawley Rats Treated with Low Doses of Aflatoxin B1 Alone or in Combination with Extremely Low Frequency Electromagnetic Fields

**DOI:** 10.3390/toxins14050325

**Published:** 2022-05-03

**Authors:** Andrea Vornoli, Eva Tibaldi, Federica Gnudi, Daria Sgargi, Fabiana Manservisi, Fiorella Belpoggi, Francesco Tovoli, Daniele Mandrioli

**Affiliations:** 1 Cesare Maltoni Cancer Research Center, Ramazzini Institute, Via Saliceto 3, 40010 Bentivoglio, Italy; andreaw86@hotmail.it (A.V.); gnudif@ramazzini.it (F.G.); sgargid@ramazzini.it (D.S.); fabiana.manservisi@libero.it (F.M.); belpoggif@ramazzini.it (F.B.); mandriolid@ramazzini.it (D.M.); 2 Division of Internal Medicine, Hepatobiliary and Immunoallergic Diseases, IRCCS Azienda Ospedaliero-Universitaria di Bologna, 40138 Bologna, Italy; francesco.tovoli2@unibo.it

**Keywords:** toxicant associated fatty liver disease, Aflatoxin b, Sprague-Dawley rats, hepatotoxicity, liver

## Abstract

The term toxicant-associated fatty liver disease (TAFLD) has been proposed to describe fatty liver diseases connected to toxicants other than alcohol. Aflatoxins are mycotoxins commonly found as contaminants in foods and feeds, which are known liver toxicants and potential candidates as potential causes of TAFLD. Aflatoxin B1 (AFB1) was administered at low doses to Sprague-Dawley (SD) rats, alone or in combination with S-50 Hz an extremely low frequency electromagnetic field (ELFEMF), to study the evolution of TAFLD, preneoplastic and neoplastic lesions of the liver and the potential enhancing effect of lifespan exposure to ELFEMF. Steatosis, inflammation and foci of different types were significantly increased in both aflatoxin-treated males and females, which is consistent with a pattern of TAFLD. A significant increase in adenomas, cystic dilation of biliary ducts, hepatocellular hyperplasia and hypertrophy and oval cell hyperplasia were also observed in treated females only. The administration of low doses of AFB1 caused TAFLD in SD rats, inducing liver lesions encompassing fatty infiltration, foci of different types and adenomas. Furthermore, the pattern of change observed in preneoplastic liver lesions often included liver steatosis and steatohepatitis (TASH). ELFEMF did not result in any enhancing or toxic effect in the liver of SD rats.

## 1. Introduction

The liver represents the main and most complex organ for metabolism, with hepatocytes playing a crucial role in ammonia and amino acids metabolism, biochemical oxidation reactions and detoxification of a variety of drugs, hormones, vitamins and environmental toxicants. Being the organ that is most involved in the first line of defense, the liver also represents the most common target organ damaged by chemicals. Among the pathologies affecting the liver, nonalcoholic fatty liver disease (NAFLD) is currently the most common in humans and is the second leading cause of primary liver cancer. In one meta-analysis, the global prevalence of NAFLD diagnosed by imaging was reported to be 25.24%. The highest prevalence was in the Middle East (37.19%) and South America (30.45%), followed by Asia (27.17%), North America (24.13%), Europe (23.17%) and Africa (13.48%) [1]. NAFLD is a condition characterized by fat accumulation in the liver (defined by a hepatic lipid content >5%), which is not attributable to alcohol consumption. When NAFLD is histopathologically characterized by the presence of both hepatic steatosis and inflammation with hepatocyte injury (ballooning), with or without fibrosis, it is defined as nonalcoholic steatohepatitis (NASH), which is mostly associated with obesity [2]. In 2010 Cave et al. [3] proposed for the first time the use of the term toxicant-associated steatohepatitis (TASH) when they observed a high prevalence of steatohepatitis in liver biopsies from highly exposed chemical workers that presented with the same pathological and morphological characteristics of NASH. In particular, the 80% of biopsied vinyl chloride (VC) workers were compatible with a diagnosis of steatohepatitis, resulting in the highest percentage of steatohepatitis ever observed in any single risk group. For example, in heavy drinkers and in bariatric surgery patients the incidence of steatohepatitis was reported to be 10–35% and 25–55%, respectively [4,5]. In the study by Cave et al. [3], the affected workers were not obese, and their steatohepatitis could not be explained by ethanol intake. Consequently, in that study sample there were no additional risk factors for steatohepatitis other than exposure to the industrial chemical VC [3]. Interestingly, VC was also one of the first chemicals to be recognized as carcinogenic for the liver, almost 50 years ago in our experimental animal model [6]. After these first observations, many classes of chemicals, already proven to be carcinogenic in humans and animal models, have been proposed to be associated with TAFLD [7]. More research is needed to understand the path from TAFLD to liver cancer induced by toxicants other than alcohol, so that more effective diagnostics, preventive and therapeutic measures may be developed. Among these, aflatoxins deserve a special role, as their carcinogenicity has been demonstrated in both humans and experimental animals. Aflatoxins are secondary metabolites, members of a group of fungal mycotoxins produced by Aspergillus flavus and *Aspergillus parasiticus*, occurring as a contaminant in a variety of foods and feeds, such as crops, cereals, oilseeds, tree nuts and spices. The B1 type (AFB1) is one of the most toxic and has mutagenic, immune-suppressive and carcinogenic effects in both humans and animals. Waterborne transmission and contamination with this poisonous mold represent a matter of concern for the agricultural field and aquatic life stock. A study by Dey et al. (2020) indicated that AFB1 contamination triggers the programmed cell death machinery, subsequently affecting the ecosystem [8]. In humans, intake of nanograms to micrograms per day of AFB1 is mainly attributable to consumption of maize and peanuts, especially in those areas of the world with hot, humid climates, where they represent staple foods [9]. The overall body of evidence collected in the most recent Monograph induced the International Agency for Research on Cancer (IARC) to include AFB1 into Group 1 of carcinogens, in particular for its role in etiology of hepatocellular carcinoma (HCC) by inducing the formation of DNA adducts [10]. Up to 28.2% of HCC cases are globally attributed to AFB1 exposure, particularly in low- and middle-income countries [11,12]. According to EFSA, current levels of exposure to AFB1 derived from food raises health concerns [13]. AFB1-induced HCC is preceded by a sequence of distinct morphologic and histochemical changes that have been documented in several experimental animal models and in humans; the dose of the chemical is one of the main factors influencing the onset and progression of these changes during the preneoplastic phase [14]. In the present study, AFB1 was administered at a low dose to Sprague-Dawley (SD) rats to investigate the evolution of preneoplastic and neoplastic lesions in the liver. Furthermore, a group of rats was exposed to AFB1 plus S-50 Hz ELFEMF to elucidate the potential enhancing role of ELFEMF in the evolution of any lesions of the liver. In fact, this lifespan whole-body exposure study is part of the integrated project of the Ramazzini Institute (RI) for studying the effects on health of ELFEMF alone or in combination with other known carcinogens. We have previously demonstrated that in our experimental rat model, life-span exposures to continuous and intermittent ELFEMF, when administered alone, did not represent a significant risk factor for cancer onset [15]. However, when administered in combination with formaldehyde or γ-radiation, ELFEMF seemed to enhance their carcinogenic potential and the development of various malignant tumors [16,17]. These findings deserve special attention in terms of public health, since exposure to ELFEMF in industrialized countries always combines with concurrent exposures to other chemical/physical agents, at low or high doses, both in the workplace and in the general environment.

## 2. Results

### 2.1. Food and Water Consumption, Body Weight and Survival

**Figure 1:**
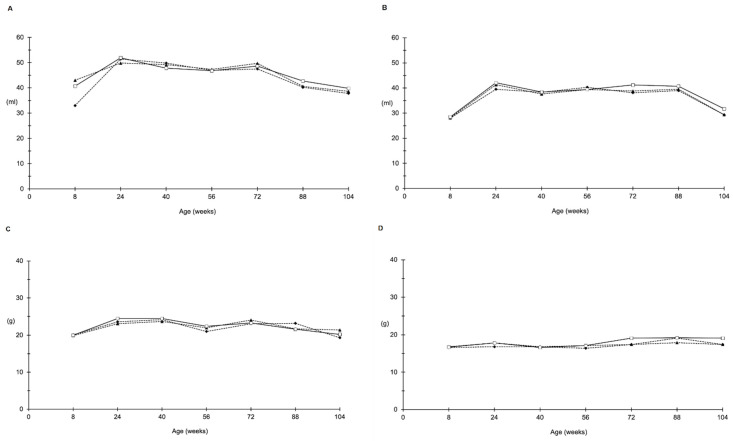
Food and water consumption. Male (**A**) and female (**B**) water consumption and male (**C**) and female (**D**) food consumption from 8 to 104 weeks of age. Data shown refer to control group (□), AFB1 (▲) and S-50 Hz ELFEMF + AFB1 (⬥) treated group.

**Figure 2:**
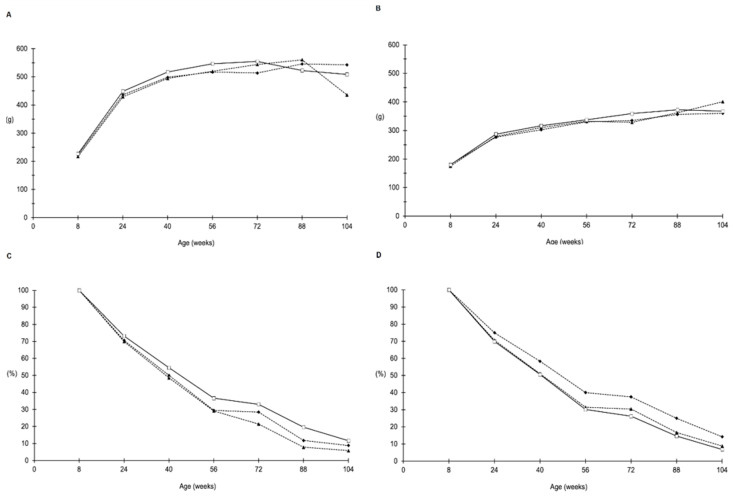
Body weight and survival. Male (**A**) and female (**B**) mean body weight from 8 to 104 weeks of age; male (**C**) and female (**D**) survival index from 0 to 104 weeks of age. Data shown refer to control group (□), Aflatoxin B1 (▲) and S-50 Hz ELFEMF + AFB1B1 (⬥) treated group.

The experiment proceeded as planned and no alteration of the clinical status of the animals was observed in any of the different groups. In-life parameters for all groups are presented in Figure 1 and Figure 2. No differences were observed in mean water consumption (Figure 1A,B), food consumption (Figure 1C,D), mean body weight (Figure 2A,B) or survival index (Figure 2C,D), either in male or in female rats.

### 2.2. Necropsy

No macroscopically detectable pathological differences among treated groups and controls were observed during interim or final sacrifices.

### 2.3. Synergistic Effects of AFB1 and ELFEMF in Male Rats

Analyses of histopathological findings did not show differences between groups during the first seven interim sacrifices. In the eighth interim sacrifice (72 weeks after the end of treatment with AFB1) some increases in the incidence of lesions compared to control were observed, none of which were statistically significant. Steatosis was observed in 10% of control animals, in 40% of animals treated with AFB1 and S-50 Hz ELFEMF and in 30% of animals treated with AFB1 only. Inflammation was observed in 20% of animals treated with AFB1 only, compared to the control group (0%). Fibrosis was not observed in controls, while it was present in 20% of the animals treated with AFB1 only, and in 10% of those treated with both AFB1 and S-50 Hz ELFEMF. Finally, and similarly, foci of any type (eosinophilic, clear cells, basophilic) were not observed in the control group, while they were present in 10% of animals treated with both AFB1 and S-50 Hz ELFEMF and in 30% of those treated with AFB1 only (Table 1).

For animals at final sacrifice and those which died during the study, the incidence of steatosis was similar in controls and animals treated with AFB1 only (16.1 and 16.7%, respectively), while the incidence of steatosis more than doubled in animals treated with both AFB1 and ELFEMF (35.3%). Thirteen percent of animals in the control group had intraparenchymal inflammation, while treated animals faced a two-fold increase (22.2% in animals treated with AFB1 only, 29.4% in those treated with both AFB1 and ELFEMF). Hepatocyte necrosis was observed twice as often in treated animals as in controls (in 16.1, 33.3 and 35.3% of animals from the control, AFB1 and AFB1 and ELFEMF groups, respectively). The increase in the incidence was even more marked for foci, which was observed in 3.2% of the controls, 16.7% of the animals treated with AFB1 only and 23.5% of those treated with both AFB1 and ELFEMF; in this case, the difference between the latter group and controls was statistically significant (two-tailed exact Fisher test, *p* = 0.047). One hepatocellular adenoma was observed in animals treated with both AFB1 and S-50 Hz ELFEMF (Table 2).

No significant differences were observed between animals treated with AFB1 alone or animals treated with ABF1 and ELFEMF. Therefore, again to increase the statistical power of our analyses, we decided to consider them as one group, with a total number of 35 males. All statistical analyses were therefore repeated, firstly by comparing the group of all treated male rats with the controls of the same experiment, and then with the 500 animals from the control group of the entire study. The results of these analyses are summarized in Table 3.

### 2.4. Synergistic Effects of AFB1 and ELFEMF in Female Rats

In females, no significant statistical differences were observed during the first seven interim sacrifices. In the eighth interim sacrifice (72 weeks after the end of treatment with AFB1) some increases in the incidence of lesions compared to control were observed (Table 4). Steatosis was observed in 20% of control animals, in 40% of animals treated with both AFB1 and S-50 Hz ELFEMF, and in 10% of those treated with AFB1 only. Inflammation was observed in 60% of animals treated with AFB1 only and in 40% of animals treated with both AFB1 and S-50 Hz ELFEMF, compared to controls. Fibrosis was not observed in controls nor in animals treated with AFB1 only, while it was present in 20% of those treated with both AFB1 and S-50 Hz ELFEMF. Foci of any type (eosinophilic, clear cells, basophilic) were not observed in the control group, while they were present in 30% of animals treated with both AFB1 and S-50 Hz ELFEMF as well as in 30% of those treated with AFB1 only. Hepatocellular adenoma was only observed in 20% of the animals treated with both AFB1 and S-50 Hz ELFEMF. The only HCC found in the whole study (Table 4) was also observed in the same group.

The analysis of data concerning animals from the final sacrifice and those spontaneously dead highlighted interesting results, as well. A non-significant increase in the incidence of steatosis was observed in animals treated with AFB1 or both AFB1 and ELFEMF (19% and 28.2%, respectively), compared to controls (13%). A significant increase in the incidence of intraparenchymal inflammation was observed in animals treated with AFB1 or both AFB1 and ELFEM (52.4% and 25.6%, respectively), compared to controls (17.4%). The differences between the two treatment groups, and between AFB1 treatment and controls was statistically significant (Chi2 test, *p* = 0.038 and Fisher Exact test, *p* = 0.025, respectively). This result is similar to what was observed when considering any type of inflammation, as showed in Table 5. Hepatocyte’s necrosis was observed three times as often in treated animals as in controls (in 8.7, 28.6 and 25.6% of animals from the control, AFB1 and AFB1 and ELFEMF groups, respectively). The increase in the incidence was again even more marked for foci, that were not observed in controls but appeared in 28.6% of the animals treated with AFB1 only, and 23.1% of those treated with both AFB1 and ELFEMF; the difference with both treatment groups and controls was statistically significant (two-tailed exact Fisher test, *p* = 0.008 and 0.020, respectively). Eosinophilic foci were by far the most common type of observed lesions, and the difference in the incidence are equally statistically significant. Five hepatocellular adenomas were observed in animals treated with both AFB1 and S-50 Hz ELFEMF (12.8% of animals, 0% in the other groups) (Table 5).

Performing 2 × 2 tests for all groups against the others showed that in just one case (inflammation) statistically significant differences in the incidence of a lesion was observed between the two treatment groups (Aflatoxin B1 and ELFEMF and AFB1 alone). Similar to how data were handled for males, all females were considered as a single group (total of 60 animals).

All statistical analyses were therefore repeated, comparing the group of all treated female rats, with the controls of the same experiment, first, and then with the 501 animals from the whole study’s control group. Table 6 summarizes the results of these analyses.

### 2.5. Pre-Neoplastic, Neoplastic and TAFLD Lesions in Liver of AFB1 Treated Male Rats

In all the lesions of interest, increases were observed in the treated animals compared to the original control group, although not statistically significant. In particular, the highest increases were observed in the incidence of steatosis, intraparenchymal inflammation, hepatocyte necrosis, foci, fibrosis and hepatocyte hypertrophy; hepatocellular adenomas and hyperplasia of the hepatocytes and of oval cells appeared more frequently, too. The comparison of the treated group with the larger common control group allowed to appreciate how some lesions that could be observed in treated animals, can be really considered quite rare, for example, foci, hepatocellular adenoma, fibrosis and hyperplasia or hypertrophy of the hepatocytes. The tests conducted against this more numerous control group resulted in some statistically significant cases: the incidence of all types of steatosis more than doubled in treated males (25.7% vs. 11.6 in controls, significant by Chi-squared test, *p* = 0.015). Hepatocyte hypertrophy was observed in 5.7% of the treated vs. 0.8 of the control animals, nearly statistically significant (*p* = 0.053). A significant increase in hepatocyte necrosis was observed in treated animals (34.3%), compared to controls (18.2%) (*p* = 0.020). The incidence of foci in general increased dramatically (20.0% vs. 1.2% in whole-study controls, significant by Fisher Exact test, *p* = 0.000); the same holds for the subtypes, in particular, for clear cell focus and eosinophilic focus (both observed in 11.4 of the observed animals and in 0.4 and 0.8% of the controls, respectively; *p* = 0.000 and 0.001 by Fisher Exact test, respectively) (Table 2).

### 2.6. Pre-Neoplastic, Neoplastic and TAFLD Lesions in Liver of AFB1 Treated Female Rats

**Figure 3:**
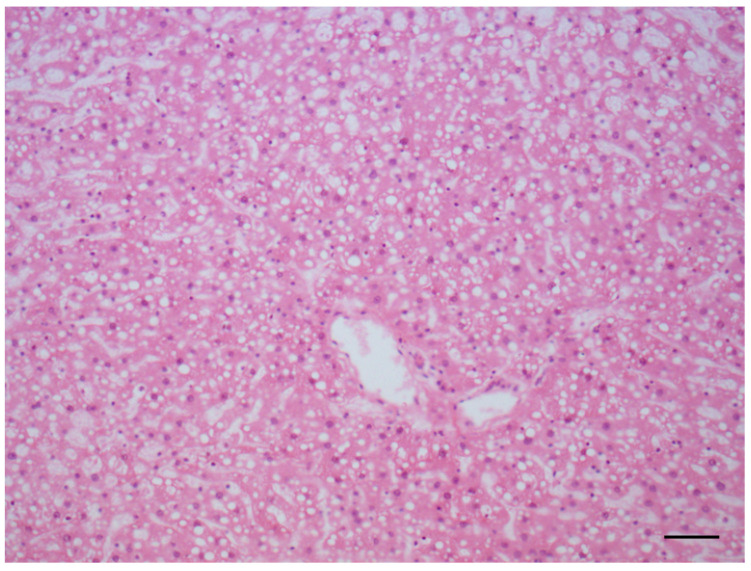
Steatosis in liver (AFB1 + ELFEMF treated female SD rat). The lesion is characterized by a mixture of fatty change (micro- and macrovesicular steatosis) (HE, 200×; scale bar 1 cm = 50 µm).

**Figure 4:**
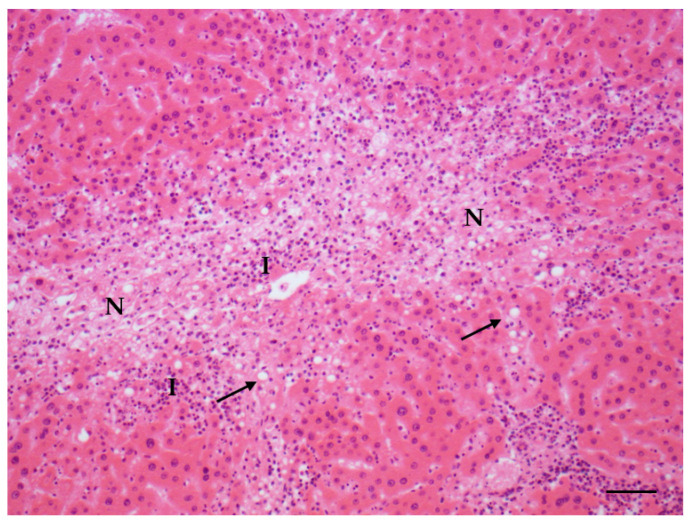
Steatosis (arrows) associated with inflammation (I) and necrosis (N) in liver (AFB1 + ELFEMF treated female SD rat) (HE, 200×; scale bar 1 cm = 50 µm).

**Figure 5:**
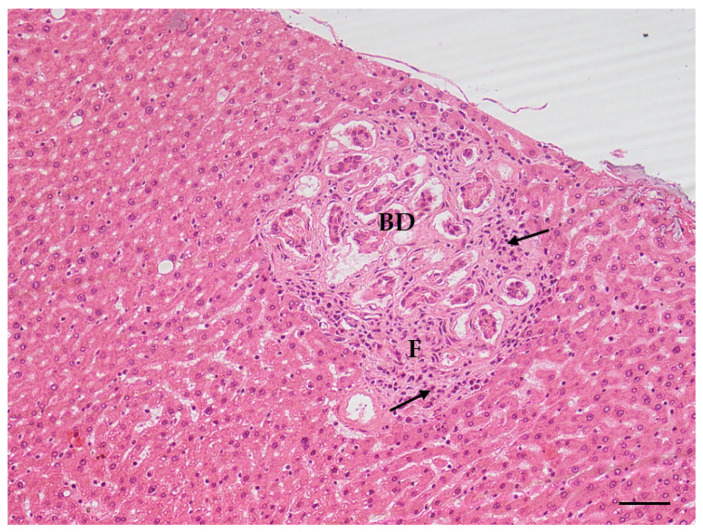
Cholangiofibrosis, bile duct hyperplasia (BD) and inflammation in liver (AFB1 + ELFEMF treated female SD rat). Early fibrosis (F) and inflammatory cell infiltration (arrows) are observed around the biliary ducts (HE, 200×; scale bar 1 cm = 50 µm).

**Figure 6:**
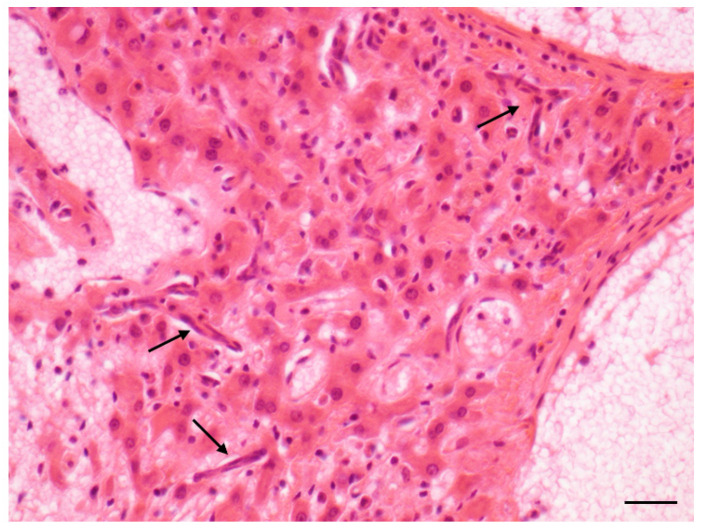
Oval cell hyperplasia in liver (AFB1 + ELFEMF treated female SD rat). The proliferating oval cells are spindloid and originates from portal areas (arrows). The lesion consists of a single row of cell along sinusoids (HE, 400×; scale bar 1 cm = 20 µm).

**Figure 7:**
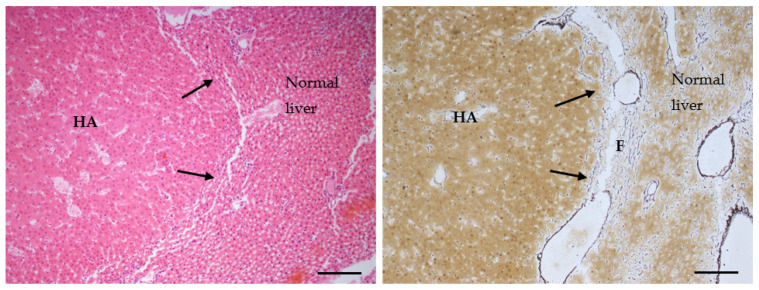
Hepatocellular adenoma (HA) (AFB1- treated female SD rat). (**Left**) Nodular lesion with compression of adjacent normal hepatocytes. The liver plates impinge obliquely on surrounding liver parenchyma (arrows) (HE, 100×; scale bar 1 cm = 100 µm). (**Right**) The silver impregnation staining (SS) helps in the assessment of the architecture of the hepatic plates, such as expansion of the hepatocytes in the nodular lesion, that is clearly distinguishable from the surrounding normal hepatic parenchyma. Fibrous encapsulation (F) and compression of the sinusoids in the hepatocellular adenoma were also observed (SS, 100×; scale bar 1 cm = 100 µm).

Increases in the incidence of all the lesions of interest were observed in the treated animals with respect to the original control group: as for males, the highest increases were observed in the incidence of steatosis (Figure 3), inflammation and hepatocyte necrosis (Figure 4), cholangiofibrosis (Figure 5), cystic dilation of the biliary ducts, hyperplasia of the hepatocytes and of the oval cells (Figure 6) and hypertrophy of the hepatocytes, hepatocellular adenoma (Figure 7).

**Figure 8:**
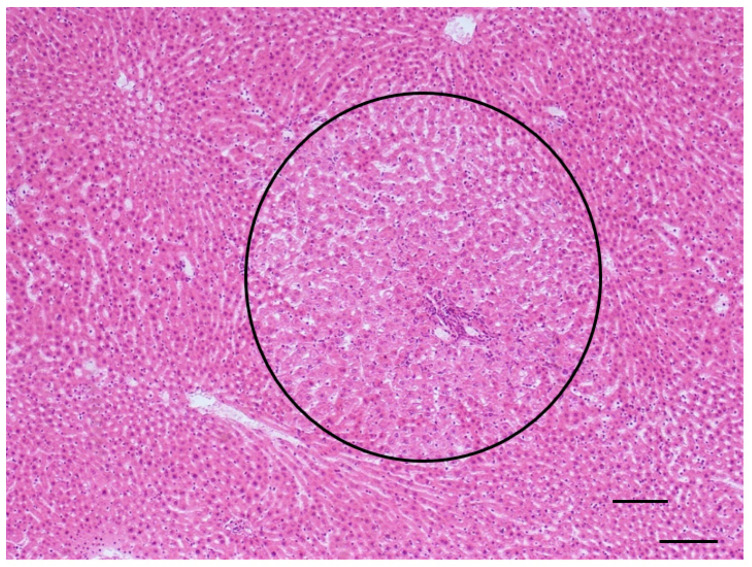
Eosinophilic focus in liver (AFB1 + ELFEMF treated female SD rat). The lesion is characterized by enlarged, polygonal hepatocytes with acidophilic staining cytoplasm. Cytoplasm is distinctly granular and pale pink, intensely eosinophilic (circle) (HE, 100×; scale bar 1 cm = 100 µm).

Statistically significant increase in the incidence of foci of any type (25% in treated animals vs. 0 in controls, significant by Fisher Exact test, *p* = 0.008), and in particular of eosinophilic foci (23.3% in treated animals vs. 0 in controls, significant by Fisher Exact test, *p* = 0.008) was observed (Figure 8 and Table 7).

**Figure 9:**
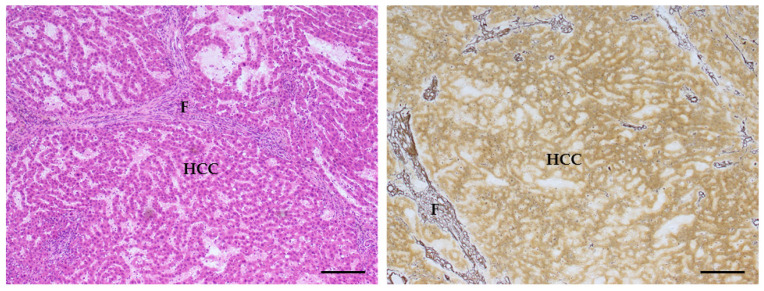
Hepatocellular carcinoma (HCC) (AFB1 + ELFEMF treated female SD rat). (**Left**) The lesion, composed of well-differentiated hepatocytes forming trabeculae of multiple cell layer, shows a local infiltrating growth and lack of distinct demarcation. The neoplastic cells are characterized by the alteration of the cytoplasmic pattern staining with increased basophilia relative to normal hepatocytes (HE, 100×; scale bar 1 cm = 100 µm). (**Right**) The silver impregnation staining (SS) highlights to a greater extent the loss of the normal lobular architecture, with irregular growth pattern, in the malignant neoplastic lesion. The presence of strong reactive reticular fibrosis (F) around the neoplastic mass and the compression of the sinusoids adjacent to the mass are also observed. (SS staining, 100×; scale bar 1 cm = 100 µm).

The comparison of the treated group with the larger common control group confirmed what was already observed for males: that lesions such as foci, hepatocellular adenoma, cystic dilation of the biliary ducts or hyperplasia or hypertrophy of the hepatocytes which were found in treated animals are very rarely observed in controls. The tests conducted against this more numerous control group resulted in some statistically significant cases: the incidence of all types of steatosis increased by four times in treated females (42.9% vs. 10.8 in controls, significant by Chi-squared test, *p* = 0.003). In particular, the most important difference was observed in the incidence of focal steatosis (22.9% vs. 2.8% in whole-study controls, significant by Chi-squared test, *p* = 0.001). Other lesions that appeared very rarely in the larger common control group were cystic dilations of the biliary ducts, hyperplasia and hypertrophy of the hepatocytes (0.6, 0.4 and 0.4% of the females, respectively); hepatocyte hypertrophy was observed in 13.3% of the treated animals, leading to a significant difference by Fisher exact test, *p* = 0.000. Hyperplasia of the hepatocytes was observed in 5% of the treated animals, so that the difference in incidence was statistically significant by Fisher exact test, *p* = 0.010. Cystic dilation of the biliary ducts was present in 8.3% of the exposed animals, again leading to a significant difference by Fisher exact test, *p* = 0.001. Furthermore, hyperplasia of the oval cells was observed in 13.3% of animals from the treated group, and in 2.4% of the controls, therefore the difference was statistically significant by Chi-squared test, *p* = 0.000. The incidence of foci in general increased even more dramatically than in males (25% vs. 0.8% in whole-study controls, significant by Chi-squared test, *p* = 0.000); the same holds for the subtypes, in particular for eosinophilic foci (observed in 23.3 of the observed animals and in 0.2% of the controls, *p* = 0.000). Hepatocellular adenomas (one or multiple) with different patterns (Figure 8) were observed in only 0.6% of the animals from the whole-study control group, and in 8.3% of the treated animals; the increase was statistically significant using Fisher Exact test, *p* = 0.001 (Table 7). In one case of a treated female, an HCC was also observed (Figure 9).

## 3. Discussion

Our previous studies showed that when administered in combination with other known carcinogens such as formaldehyde or γ-radiation, ELFEMF enhanced carcinogenic potential and the development of various malignant tumors [16,17]. In the present study, the concurrent exposure to AFB1 and ELFEMF did not induce any statistically significant increased hepatotoxic or carcinogenic effects, both in male and female SD rats. Furthermore, no difference was observed between the two treatment groups (AFB1 alone and AFB1 + ELFEMF) in any of the parameters analyzed in the present study. The consequent decision to consider the two treatment groups as a single group enabled an increase in the statistical power of our analyses to better understand the effects due to AFB1 administration as compared to both concurrent controls and the larger BT1CEM-common control group. AFB1 ingestion is well known to cause HCC, being an aflatoxin classified as a Group 1 carcinogen according to IARC [10]; however, can intake of low doses of AFB1 be the cause of TAFLD/TASH upstream of HCC? Here, we investigated the evolution of steatogenic, preneoplastic and neoplastic lesions in the liver of rats, which were not obese or exposed to alcohol, which were treated with very low doses of AFB1. For a long time, the accumulation of lipids in hepatocytes has been considered a benign toxicologic finding, but the increasing association between HCC and the current steatohepatitis epidemic inevitably led to a reassessment of the significance of fatty liver diseases due to environmental and industrial toxicants. Fatty liver is now considered the most common pathologic hepatic response to toxicant exposure [3,7]. In our experimental conditions, the incidence of steatosis was increased in all groups treated with AFB1; steatosis focal and total (as the summa of focal and diffuse) in females and steatosis total in males resulted in a significant increase in AFB1-treated animals compared to BT1CEM control group. Previous rat studies have associated the exposure to different doses of AFB1 to lipid accumulation in the liver. In 2011, Zhang et al. demonstrated that twelve days of treatment with a moderate/intermediate dosage of AFB1 (0.32 mg/kg body weight/day) induced formation of hepatic vacuole and lipid droplets in SD rats, thus indicating that AFB1 exposure caused hepatic steatosis [18]. Lipid accumulation in the periportal hepatocytes was also previously observed in SD rats exposed to a much higher dosage AFB1 (3 mg/kg) with in vivo MRI assessment [19]. Mitochondrial malfunction, impaired fatty acid exportation, increased cytokine production and insulin resistance were suggested as possible mechanisms for the development of steatosis by chemical toxicants [20]. Questions remain concerning the potential causal relationship between exposure to toxicant and the development of fatty liver disease. Treviño and Katz in a recent review suggested a possible linkage between endocrine disruptors and fatty liver disease in humans via modulation of xenobiotic nuclear receptors [21]. AFB1 endocrine disrupting properties has just been explored recently [22,23,24]. In addition, nuclear receptors seem to play a crucial role in the pathogenesis of TAFLD: activation of nuclear receptors following xenobiotic exposure results in the induction of phase I and phase II drug-metabolism enzymes. Specifically, the peroxisome proliferator activated receptors (PPARs), the pregnane X receptors (PXR), the constitutive androstane receptor (CAR), the liver X receptor (LXR), the farnesoid X receptor (FXR) and acyl hydrocarbon receptor (AHR) have been shown to participate in non-alcohol fatty liver disease induction [20,21]. Similarly to the other forms of fatty liver, the transition of the disease in TASH could be determined by a progressive “two hit model”, with the “(multiple) secondary hits” occurring on the background of lipid deposition and consisting of the increase in oxidative stress, inflammatory cytokines, insulin resistance and mitochondrial dysfunction which produce inflammatory infiltration, fibrosis and hepatocytes necrosis typical of the steatohepatitis. Evidence in both humans and animal models have shown the negative impact of AFB1 in the normal cellular physiology and activities of immune cells of living organisms by both inducing oxidative stress and stimulating cytokines production [25]. Two recent studies showed the significantly higher secretion of inflammatory cytokines in the liver and colon of mice exposed to AFB1, when compared to their respective controls [26,27].

An enhancement in the incidence of these above-described peculiar TASH histopathological findings in both males and females has been observed in our AFB1-treated animals when compared to controls. However, the triggers initiating the process remain undefined [28,29,30,31]. Apoptosis and necrosis have been shown to coexist in human ASH and NASH, with apoptosis tending to be more dominant in NASH and necrosis in ASH. Differently, hepatocyte necrosis was prevalent to apoptosis in TASH [32]. Several experimental studies have shown that the earliest distinct phenotypic parenchymal change indicating carcinogenic response in chemical hepatocarcinogenesis is represented by foci of altered hepatocytes (FAH), whose phenotype share great similarities across species. The detection of FAH with a similar phenotype in the livers of rodents and humans favor the extrapolation of data from animals to humans and result of particular interest for human secondary prevention of HCC, the development of which has been estimated to take over 30 years [33]. In our experiment, a statistically significant increase in the incidence of preneoplastic FAH has been reported for both sexes of rats exposed to AFB1. In particular, two different types of FAH were observed by us: eosinophilic and clear cell ones. FAH have been shown to precede both adenomas and carcinomas in the liver, representing the most prevalent form of hepatic preneoplasia observed in animals for a long time, and identified in human chronic liver diseases associated with, or predisposing to, hepatocellular carcinomas more recently. Morphological, microbiochemical and molecular biological approaches in situ revealed striking similarities in specific changes of the cellular phenotype of preneoplastic FAH developing in experimental and human hepatocarcinogenesis, irrespective of whether this was elicited by chemicals, hormones, viruses or radiation. The advantage of using FAH for risk identification (aiming at primary cancer prevention) in long-term and medium-term carcinogenesis bioassays has been well documented, but quantitative morphometric approaches appear to be indispensable for an appropriate evaluation of both bioassays. The detection of phenotypically similar FAH in various animal models and in humans prone to develop or bearing hepatocellular carcinomas favors the extrapolation from data obtained in animals to humans. In a study by Qian et al. [34], an integrative toxicopathological evaluation of AFB1 exposure demonstrated that FAH, other than being sensitive biomarkers for AFB1 toxic effect, correlated with proliferation of cells of the bile duct epithelium and oval cell in F344 rats. The same effect was observed in our treated female SD rats showing a significantly increased incidence of oval cell hyperplasia and cystic dilation of the biliary duct, when compared to controls; moreover, an increase in these parameters, although not significant, was also observed in male rats. The only HCC observed in the whole study was found in a treated female rat of the eighth sacrifice. On the other hand, our experimental conditions determined an increased incidence of hepatocellular adenoma both in male and female rats. There was no evidence of cirrhosis, not even in those animals in which adenomas were observed, a point of some importance since AFB1 is associated with cirrhosis in humans. However, it is not that surprising to us since our strain is more prone to developing localized fibrosis than cirrhosis. The liver is the predominant site for AFB1 metabolism, which in turn represents the main cause for its toxic mechanism [35]. In rodents, the same receptors involved in the pathogenesis of TAFLD are activated by xenobiotics in the formation of liver tumors [20]. As a consequence, AFB1 metabolization mostly occurs upon the action of the members of the P450 superfamily of microsomal enzymes (CYPs). The key event in the genotoxic process leading to AFB1-mediated hepatocarcinogenesis is represented by the epoxidation of AFB1 by CYPs, culminating with the transformation into the final carcinogen Aflatoxin B1-8,9-epoxide (AFBO), which is characterized by two isomers: exo-8,9-epoxide and, to a lesser extent, endo-8,9-epoxide [36]. Being highly electrophilic, AFBO is naturally capable of forming adducts to amines promoting mutations in the nucleotide sequence, thus interrupting the function of biological molecules involved. In particular, the exo isomer has a greater affinity for DNA than the endo isomer and has its highest concentration in the liver, so that it is considered the major hepatocarcinogenic metabolite [37]. Microsomal biotransformation of the toxin also includes hydroxylation of AFB1, another mechanism potentially exerting toxic effects. Among the hydroxylated metabolites, the most carcinogenic is represented by AFM1 which, similarly to AFBO, is capable of forming an adduct with the DNA. CYP3A4 and CYP1A2 are the most active isoenzymes of this family to activate AFB1. At low AFB1 concentration, the main producer of the exo isomer of AFBO is CYP1A2, which is also responsible for the formation of hydroxylated AFM1, thus resulting in the isoform of CYP most involved in AFB1-mediated hepatocarcinogenesis [38]. Of primary importance for understanding the mechanism behind nucleic damage are also epigenetic changes, whose role is crucial in AFB1-induced carcinogenesis [39]. Three kinds of modifications are included in epigenetic alterations: DNA methylation, histone modifications and regulation of noncoding RNA (such as miRNA). These modifications may be the cause of the development of carcinogenesis through different alterations such as chromosomal instability, oncogene activation and tumor suppressor gene inactivation [40].

## 4. Conclusions

With the present experimental study, we have explored the hepatic effects of AFB1, administered to SD rats at a low dose, over time. The treatment determined lesions were consistent with a pattern of TAFLD-TASH. In some cases, the pathology progressed to preneoplastic FAH or hepatic neoplasms, in particular hepatocellular adenomas. ELF-EMF did not determine any enhancing or toxic effect. The association of AFB1 with the development of TAFLD/TASH enables subsequent mechanistic animal studies and clinical translation in exposed human. In fact, AFB1 contamination remains an unneglectable threat to public health, not only in developing countries. Furthermore, potential synergistic effects with other environmental toxins should be taken into account, since multiple exposure at low doses is probable in light of the prolonged periods of drought and floods we are experiencing nowadays. Overall, our study gives a new insight on the progressive liver damage induced by AFB1, that for the first time encompasses the concept of TAFLD.

## 5. Materials and Methods

### 5.1. Treatment and Conduct of the Experiment

Two groups of male and female SD rats, consisting of 427 animals in total, were treated with 70 µg/rat AFB1 dissolved in 150 µL dimethyl sulfoxide (DMSO), administered by gavage, nine times in two weeks between the sixth and eighth week of age. The AFB1 administration protocol and doses were based on a previous experiment by Soffritti et al. [14]. Of these rats, one group of 222 animals had also been exposed to ELFEMF at 1000 μT of S-50 Hz MF. Treatment with S-50 Hz ELFEMF began during embryonic life irradiating the female breeders (dams) from the 12th day of pregnancy, and for the offspring it lasted until natural death. Throughout the study, daily duration of the exposure lasted 19 h/day. The exposure system was deactivated (switched off) for 5 h a day to allow clinical observation of animals, and room and animal cleaning. A total of 215 control rats were not exposed to ELFEMF, and received the vehicle (DMSO) only, without AFB1, by gavage. The experimental plan is summarized in Table 7. Ten rats/sex/group were subjected to 8 interim sacrifices between 10 and 80 weeks of age (at 2, 6, 10, 14, 22, 32, 42, 72 weeks after the end of treatment with AFB1), while the remaining animals were sacrificed at 118 weeks of age (109 weeks after the end of the treatment with AFB1). AFB1 was supplied as a powder by Sigma Aldrich S.r.l. (Milan, Italy) at a purity level (HPLC) ≥ 98.00%. For administration by gavage to experimental animals, AFB1 was dissolved in dimethyl sulfoxide (DMSO), also supplied by Sigma Aldrich S.r.l. (Milan, Italy). The ELFEMF exposure system is described in our previous papers [16,17].

### 5.2. Diet

The animals were fed ad libitum with standard feed in pellets purchased from the “Laboratorio Dottori Piccioni” (Milan, Italy), whose formulation is certified for each supply used at the Cesare Maltoni Cancer Research Center of the RI (CMCRC/RI) over a period of more than 40 years. Animals were administered with tap water ad libitum. Periodical analyses allowed us to keep feed and water monitored for the presence of any contaminants.

### 5.3. Experimental Animals

Animals were obtained from the colony of SD rats of the *Cesare Maltoni* Cancer Research Center of the Ramazzini Institute (CMCRC/RI), the same strain used for over 40 years, with a basic expected tumorigram and its fluctuations based on data extrapolated from more than 20,000 historical controls. The procedure for the generation of experimental rats followed the previously described Standard Operating Procedures (SOP) of the CRCCM/RI [16]. Male and female breeders were all euthanized by CO_2_ overexposure, respectively, 3 weeks after birth of offspring and 1 week after their weaning (4–5 weeks of age). The experimental rats were identified by ear punch (Jackson Laboratory method) and distributed by sex, litter by litter, until the planned number was reached for each group. After weaning, animals received standard feed and water ad libitum and were housed 5 per cage, in polycarbonate cages (41 × 25 × 15) and a shallow layer of white wood shavings as bedding. A controlled temperature of 22 ± 3 °C and a relative humidity of 40–60%, with 12 h/daylight/dark alternation were guaranteed for the entire duration of the experiment to keep all experimental animals in a controlled, homogeneous environment. The animals were kept under observation until each respective interim sacrifice, while moribund animals were sacrificed to avoid unnecessary sufferings. Starting from 6 weeks of age, the mean daily drinking water and feed consumptions were measured from each group, in a sample of 100 animals per group (50 males and 50 females) every 2 weeks for the first 8 weeks, and then every 4 weeks for 110 weeks. Status and behavior of animals were examined three times a day and clinical examination for gross changes was made every 2 weeks for the first 8 weeks and every 4 weeks until the end of the experiment. The animals were weighed every 2 weeks for the first 8 weeks from 6 weeks of age, and then every 4 weeks for 110 weeks, and then every 8 weeks until the end of the experiments. The experiment was conducted according to the current (2001–2004) Italian law regulating at the time, the protection of animals used for experimental and other scientific purposes (Decreto Legislativo 116, 1992). The experiment was performed following the principles of Good Laboratory Practice (GLP), with the same SOP as in the other concurrent studies from our comprehensive experimental project on ELFEMF [15,16,17].

### 5.4. Histopathology

After death, all experimental animals were subjected to complete necropsy following laboratory SOPs. The tissues and organs collected were preserved in a 70% solution of Solvanol (a mixture of ethyl and isopropyl alcohol, respectively, approximately 60% and 40%, obtained from Vital srl, Bologna, Italy), and 30% distilled water, except for bone tissues, which were preserved in 10% formalin and then decalcified. For liver tissue, trimming was performed according to SOPs. Each trimmed liver specimen was processed and embedded in paraffin blocks according to laboratory standard SOP. Then 3–5 μm sections were cut and routinely stained with Hematoxylin-Eosin (HE). Histopathology evaluation was systematically performed as blinded evaluation by two pathologists, without prior knowledge of the groups. A senior pathologist peer reviewed all pre-neoplastic lesions and those of oncological interest as well as any lesion of dubious interpretation. For pathological diagnoses, the same evaluation criteria and the same classification based on INHAND (International Harmonization of Nomenclature and Diagnostic Criteria for Lesions in Rats and Mice) guidelines, were adopted by all the RI pathologists [41]. A staging score was developed to assess the degree of both severity (minimal, mild or severe) and extent (diffuse or focal) of inflammatory, necrotizing or other degenerative lesions such as fatty change. The scoring system described was based on the main histological features for the diagnosis of lesions attributable to TAFLD and/or TASH in humans [42]. In fact, rodent models successfully simulate the histological patterns of the disease, even if they cannot reflect all the clinical and aetiological aspects of the human counterpart [43].

Finally, silver impregnation staining (Bio-Optica, Milan, Italy), the elective staining for the reticular connective fibers, was performed to help pathologists in the assessment of the architecture of the hepatic plates, such as expansion in regenerative and neoplastic conditions and compression of plates in nodular regenerative hyperplasia [44]. Silver staining is also a useful tool for the differential diagnosis of well-differentiated HCC and benign hepatocellular tumors such as hepatocellular adenoma. In fact, reticulin fibers are normally intact and clearly defined in benign hepatocellular tumors, even if they show cytological and structural atypia; on the contrary, the loss of reticulin framework is characteristic of HCC. Moreover, in the presence of necrosis the reticulin framework appears collapsed. All the diagnoses are reported in the experimental registries.

### 5.5. Statistical Analyses

The primary objective of statistical analyses was to identify significant differences in the incidence of TAFLD, preneoplastic and neoplastic lesions. The analysis was divided into two parts, coherently with the design of the study: the data regarding animals from the interim sacrifices, as per study design (10 males and 10 females per group, sacrificed at 2, 6, 10, 14, 22, 32, 42 and 72 weeks after the end of treatment with AFB1) analyzed separately by timepoint, on one side; on the other, the data from animals sacrificed at the final timepoint (109 weeks after the end of treatment with AFB1) and those that died before the scheduled interim sacrifice. This latter group can be considered akin to a classic carcinogenicity bioassay.

Statistical methods were chosen in accordance with the relevant guidelines [45,46] and using tests and tools that are routinely used for these purposes. For both types of data, to assess the significance of the differences in the incidence of the lesions of interest among each treatment group and the control (2 × 2 tests), a two-tailed Chi-squared test (when all expected frequencies were ≥5) or a two-tailed Fisher’s Exact test (when at least one expected frequency was <5) were used. *p* values < 0.05 were considered statistically significant. In order to increase the statistical power and the robustness of our results, 1001 additional control animals (500 males and 501 females) were considered for a second stage of analysis. These animals represented the common control group of the three additional experiments that constituted the large study on the effects of electromagnetic fields [15,16,17]. Additionally, differences in the incidence of the lesions of interest between treated animals and the added controls (2 × 2 tests) were analyzed using a two-tailed Chi-squared test (when all expected frequencies were ≥5) or a two-tailed Fisher’s Exact test (when at least one expected frequency was <5).

## Figures and Tables

**Table 1:** Incidence of specific liver lesions in male SD rats exposed to AFB1 + S 50 Hz ELFEMF, AFB1 and controls, and sacrificed in the eighth interim sacrifice (72 weeks after the end of treatment with AFB1). Results of statistical analysis on differences in incidence based on Chi-squared or Fisher Exact tests.

Sex	M								
Group (Dose)	Group I (Controls)		Group II (AFB1)			Group III (AFB1 + S 50 Hz ELFEMF)			
No	10		10			10			
	*n*	%	*n*	%	*p*-Value II vs. Ctrl	*n*	%	*p*-Value III vs. Ctrl	*p*-Value III vs. II
Inflammation	0	0.0	2	20.0	0.474	0	0.0	-	0.474
Steatosis	1	10.0	3	30.0	0.582	4	40.0	0.303	1.000
Fibrosis	0	0.0	2	20.0	0.474	1	10.0	1.000	1.000
Foci	0	0.0	3	30.0	0.211	1	10.0	1.000	0.582
Hepatocellular Adenoma	0	0.0	0	0.0	-	0	0.0	-	-
Hepatocellular carcinoma (HCC)	0	0.0	0	0.0	-	0	0.0	-	-

**Table 2:** Incidence of liver lesions in male SD rats exposed to AFB1 + S 50 Hz ELFEMF, AFB1 and controls, observed in animals at scheduled sacrifice (109 weeks after the end of treatment with AFB1) or observed in animals which died prior to scheduled sacrifice. Results of statistical analysis on differences in incidence based on Chi-squared or Fisher Exact tests.

Sex	M								
Group (Dose)	Group I (Controls)		Group II (AFB1)			Group III (AFB1 + S 50 Hz ELFEMF)			
No	31		18			17			
	*n*	%	*n*	%	*p*-Value II vs. Ctrl	*n*	%	*p*-Value III vs. Ctrl	*p*-Value III vs. II
Inflammation intraparenchymal	4	12.9	4	22.2	0.443	5	29.4	0.247	0.711
Inflammation total	5	16.1	4	22.2	0.708	5	29.4	0.295	0.711
Steatosis	5	16.1	3	16.7	1.000	6	35.3	0.163	0.264
Hepatocyte necrosis	5	16.1	6	33.3	0.286	6	35.3	0.163	1.000
Foci	1	3.2	3	16.7	0.134	4	23.5	0.047 *	0.691
Hepatocellular Adenoma	0	0.0	0	0.0	-	1	5.9	0.354	0.486

* Statistically significant difference from control group by Chi^2^/Fisher Exact test.

**Table 3:** Incidence of specific liver lesions in male SD rats exposed to AFB1 and AFB1 + S 50 Hz ELFEMF, compared to concomitant controls and to controls from the same experimental project (BT1CEM).

Sex	M							
Group (Dose)	BT1CEM Controls		Group I (Controls)		Group II + III (AFB1; S-50 Hz ELFEMF + AFB1)			
No	500		31		35			
	*n*	%	*n*	%	*n*	%	*p*-Value II + III vs. BT1CEM Ctrl	*p*-Value II + III vs. I
Inflammation intraparenchymal	81	16.2	4	12.9	9	25.7	0.146	0.192
Inflammation total	89	17.8	5	16.1	9	25.7	0.258	0.382
Steatosis	58	11.6	5	16.1	9	25.7	0.015 #	0.342
Fibrosis	6	1.2	0	0.0	2	5.7	0.091	0.494
Hepatocyte hyperplasia regenerative	3	0.6	0	0.0	1	2.9	0.134	1.000
Hepatocyte hypertrophy focal	4	0.8	0	0.0	2	5.7	0.053	0.494
Oval cell hyperplasia	10	2.0	0	0.0	1	2.9	0.528	1.000
Hepatocyte necrosis	91	18.2	5	16.1	12	34.3	0.020 #	0.158
Basophilic focus	0	0.0	0	0.0	1	2.9	0.065	1.000
Clear cells focus	2	0.4	0	0.0	4	11.4	0.000 #	0.116
Eosinophilic focus	4	0.8	1	3.2	4	11.4	0.001 #	0.360
Foci total	6	1.2	1	3.2	7	20.0	0.000 #	0.058
Hepatocellular Adenoma	2	0.4	0	0.0	1	2.9	0.060	1.000

# Statistically significant difference between rats exposed both to AFB1 and AFB1 + S50 Hz and BT1CEM control group by Chi-squared/Fisher Exact test.

**Table 4:** Incidence of specific liver lesions in female SD rats exposed to AFB1 + S 50 Hz ELFEMF, AFB1 and controls, and sacrificed in the eighth interim sacrifice (72 weeks after the end of treatment with AFB1). Results of statistical analysis on differences in incidence based on Chi-squared or Fisher Exact tests.

Sex	F								
Group (Dose)	Group I (Controls)		Group II (AFB1)			Group III (AFB1 + S 50 Hz ELFEMF)			
No	10		10			10			
	*n*	%	*n*	%	*p*-Value II vs. Ctrl	*n*	%	*p*-Value III vs. Ctrl	*p*-Value III vs. II
Inflammation total	1	10.0	6	60.0	0.057	4	40.0	0.303	0.656
Steatosis total	2	20.0	1	10.0	1.000	4	40.0	0.628	0.303
Fibrosis	0	0.0	0	0.0	-	2	20.0	0.474	0.474
Foci total	0	0.0	3	30.0	0.211	3	30.0	0.211	1.000
Hepatocellular Adenoma	0	0.0	0	0.0	-	2	20.0	0.474	0.474
Hepatocellular Carcinoma (HCC)	0	0.0	0	0.0	-	1	10.0	1.000	1.000

**Table 5:** Incidence of liver lesions in female SD rats exposed to AFB1 + S 50 Hz ELFEMF, AFB1 and controls, observed in animals at scheduled sacrifice (109 weeks after the end of the treatment with AFB1) or observed in animals that died prior to scheduled sacrifice. Results of statistical analysis on differences in incidence based on Chi-squared or Fisher Exact tests.

Sex	F								
Group (Dose)	Group I (Controls)		Group II (AFB1)			Group III (AFB1 + S 50 Hz ELFEMF)			
No	23		21			39			
	*n*	%	*n*	%	*p*-Value II vs. Ctrl	*n*	%	*p*-Value III vs. Ctrl	*p*-Value III vs. II
Inflammation intraparenchymal	4	17.4	11	52.4	0.025 *	10	25.6	0.541	0.038 °
Inflammation total	5	21.7	12	57.1	0.029 *	12	44.4	0.561	0.047 °
Steatosis total	3	13.0	4	19.0	0.693	11	28.2	0.218	0.541
Hepatocyte necrosis	2	8.7	6	28.6	0.126	10	25.6	1.000	0.182
Eosinophilic focus	0	0.0	6	28.6	0.008 *	8	20.5	0.021 *	0.532
Foci total	0	0.0	6	28.6	0.008 *	9	23.1	0.020 *	0.757
Hepatocellular Adenoma	0	0.0	0	0.0	-	5	12.8	0.152	0.148

* Statistically significant difference from control group by Chi-squared/Fisher Exact test. ° Statistically significant difference from AFB1 treatment group by Chi-squared/Fisher Exact test.

**Table 6:** Incidence of specific liver lesions in female SD rats exposed both to AFB1 and AFB1 + S 50 Hz ELFEMF, compared to concomitant controls and to controls from the same experimental project (BT1CEM).

Sex	F							
Group (Dose)	BT1CEM Controls		Group I (Control)		Group II + III (AFB1; S 50 Hz ELFEMF + AFB1)			
No	501		23		60			
	*n*	%	*n*	%	*n*	%	*p*-Value II + III vs. BT1CEM Ctrl	*p*-Value II + III vs. I
Inflammation intraparenchymal	178	35.6	4	17.4	21	35.0	0.935	0.181
*Inflammation total*	184	36.7	5	21.7	24	40.0	0.620	0.133
Steatosis diffuse	40	8.0	2	8.7	7	20.0	0.324	1.000
Steatosis focal	14	2.8	1	4.3	8	22.9	0.001 #	0.433
*Steatosis total*	54	10.8	3	13.0	15	42.9	0.003 #	0.373
Fibrosis	10	2.0	1	4.3	2	3.3	0.374	1.000
Cholangiofibrosis focal	15	3.0	1	4.3	5	8.3	0.052	1.000
Cystic dilation biliary duct	3	0.6	1	4.3	5	8.3	0.001 #	1.000
Hepatocyte hyperplasia regenerative	2	0.4	0	0.0	3	5.0	0.010 #	0.557
Hepatocyte hypertrophy focal	2	0.4	0	0.0	8	13.3	0.000 #	0.099
Oval cell hyperplasia	12	2.4	0	0.0	8	13.3	0.000 #	0.099
Hepatocyte necrosis diffuse	83	16.6	1	4.3	11	31.4	0.716	0.165
Hepatocyte necrosis focal	15	3.0	1	4.3	5	8.3	0.052	1.000
*Hepatocyte necrosis*	98	19.6	2	8.7	16	26.7	0.233	0.134
Basophilic focus	0	0.0	0	0.0	0	0.0	-	-
Clear cells focus	3	0.6	0	0.0	2	3.3	0.091	1.000
Eosinophilic focus	1	0.2	0	0.0	14	23.3	0.000 #	0.008 *
*Foci total*	4	0.8	0	0.0	15	25.0	0.000 #	0.008 *
Hepatocellular Adenoma (one)	3	0.6	0	0.0	4	6.7	0.003 #	0.572
Hepatocellular Adenoma (multiple)	0	0.0	0	0.0	1	1.7	0.107	1.000
*Hepatocellular adenoma*	3	0.6	0	0.0	5	8.3	0.001 #	0.316

# Statistically significant difference between rats exposed both to AFB1 and AFB1 + S50 Hz and BT1CEM control group by Chi-squared/Fisher Exact test. * Statistically significant difference between rats exposed both to AFB1 and AFB1 + S50 Hz and concurrent control group by Chi-squared/Fisher Exact test.

**Table 7:** Long-term bioassay of Aflatoxin B1, administered to SD rats alone or in combination with S-50 Hz ELFEMF.

Groups	Animals		Treatment	
	Sex	No	Aflatoxin B1 ^a^	S-50 Hz MF (µT) ^c^
I	M	112	0 ^b^	0
	F	103		
	M + F	215		
II	M	103	70 µg/rat	0
	F	102		
	M + F	205		
III	M	102	70 µg/rat	1000 C
	F	120		
	M + F	222		
Total	M	317		
	F	325		
	M + F	642		

^a^ Aflatoxin B1 was dissolved in DMSO at the indicated dose and administered by gavage nine times in two weeks, between the sixth and eighth week of age. ^b^ 150 µL of vehicle (DMSO) only was administered by gavage to control rats. ^c^ The treatment with S-50 Hz for 19 h/day, in continuous © mode started on the12th day of pregnancy and lasted until natural or scheduled death.

## Data Availability

All the data is contained within the present article.
